# Aneurism of the Innominate Artery

**Published:** 1882-05

**Authors:** J. S. Lynch

**Affiliations:** Professor of Principles and Practice of Medicine, and Clinical Professor of the Diseases of Heart, Throat, and Lungs


ARTICLE III.

### ANEURISM OF THE INNOMINATE ARTERY.

Clinical Lecture delivered at the College of Physicians and Surgeons, Baltimore,

BY J. S. LYNCH, M.D.,
Professor of Principles and Practice of Medicine, and Clinical Professor of the Diseases of Heart, Throat, and Lungs.
REPORTED BY T. J. SHACKLEFORD, M.D.

GENTLEMEN:—I have the pleasure to-day of bringing before you this patient, who is suffering from a tumor situated in the region of the left sterno-clavicular articulation.

This lady is thirty-five years of age, married, and by occupation a seamstress. There is nothing of clinical importance pertaining to her family history that would be of any aid to us in making our diagnosis here.

The patient was in apparently good health up to September last, when she states that while sewing at the machine she experienced a sensation of fulness in the region of the tumor, excessive sensibility of the part, vertigo, and a feeling of "faintness." She also states that at this time a physician was called in, who after treating her for a number of days, probably mistook it for a case of globus hystericus, and so informed her. As no improvement took place, she presented herself to the resident physician of the hospital, complaining of cough, difficulty in breathing, and weakness, which led him to direct my attention to the case.

I will now make the physical examination before you, which I think will convince you of the correctness of my diagnosis.

This tumor, you will observe, is located in the left infra-clavicular region at the junction of the clavicle with the manubrium of the sternum, and is of sufficient size to be seen even at a distance. Occupying as it does, so important a relation to the great blood-vessels, we are led to suspect an aneurism. There are five physical signs of aneurism here which I beg of you to remember:

1st. Presence of a tumor. 2d. This tumor pulsating. 3d. Flatness on percussion, without lung symptoms, this flatness fading into dulness bordering on healthy tissue. 4th. A bruit which has two parts, viz., (*a*) short blowing sound and (*b*) a dull muffled sound, but the two sounds are not constant. 5th. Thrill or fremitus.

Now let us see whether or not the tumor before us presents these characteristics. As to the first sign : a tumor is present beyond a doubt, as evidenced by the bulging in the position indicated ; both the second and fourth signs (pulsation and bruit) are, even to an inexperienced observer, distinguishable by auscultation and palpation in this case. By percussion we get the third sign, distinct flatness over the spherical portion of the tumor, which flatness fades into the natural percussion note as we approach the healthy tissue ; the fifth sign is also present.

Now, gentlemen, there is no doubt in my mind that this tumor is aneurismal. As to its origin : It would seem at first sight that the aneurism arises from the transverse arch of the aorta, firstly on account of the comparative frequency of aneurism at this point, and, secondly, owing to the force exerted at this point by the systolic current. But, contrary to this hypothesis, we have not the symptoms that would be produced should the tumor occupy this position. Let us see what the symptoms would be. You have but to remember the important anatomical relations in this situation to understand that the symptoms produced would be of the gravest character, many of them referable to other localities. In the posterior aspect we have the trachea, œsophagus, and thoracic duct ; the recurrent laryngeal nerve winds around the aorta on the left side, and the large arterial vessels which furnish the blood supply to the head and upper extremities make their exit at this point. Taking these anatomical relations into consideration, what would be the result? Œdema of upper extremities by pressure exerted upon the veins which return the blood from the head and upper extremities and a corresponding reduction in temperature of these parts ; spasm or paralysis of the muscles of the larynx by pressure on the recurrent laryngeal nerve. Deglutition would doubtless be endangered by pressure on the œsophagus, or inanition result from pressure on the thoracic duct ; besides this there would be a retardation in all of the pulses beyond as compared with the systole of the heart, which is not the case here. We feel safe in saying that it cannot be in the ascending arch of the aorta, for aneurisms in this situation are, as a rule, to the right of the median line, opposite to the anterior aspect of the thorax, and, owing to the close proximity to the lung of this side, would produce lung symptoms referable to that side, which symptoms are absent in the patient before you ; besides all of the pulses beyond the obstruction would be asynchronous with the systole of the heart. Should an aneurism occur in the descending arch, what would be the symptoms ? In such a case the tumor is usually situated on the left side of the vessel in a backward direction, and, by pressure exerted on the bodies of adjoining vertebra and also the ribs, would tend to cause their destruction and there is usually associated with it a more or less constant gnawing, aching, or burning pain in the region of the vertebra affected. The tumor would also cause constant and severe intercostal neuralgia, on the left side, by pressure on the intercostal nerves. It may also produce symptoms referable to pressure upon the trachea, left bronchus, œsophagus, and of the right or left lung. After taking into consideration the above symptoms, we have no hesitancy in excluding aneurism of the descending aorta.

We will now see what information we can gain from the pulse to aid us in our diagnosis.

There is one sign connected with the arterial pulse, which, when present, is an infallible guide to the location of an aneurism on any of the great vessels. This sign will rarely be met with in very small aneurisms, or at the very beginning of even large ones, but it will very generally be found whenever the aneurism has attained any considerable size. The sign I allude to is this : whenever an aneurism forms upon a vessel, it acts as a diverticulum into which the blood is poured at the heart's systole, and the aneurismal sac is first filled before the artery *beyond* the sac receives its supply. Consequently the pulse in the artery beyond the tumor will be felt *after* the pulsation in the tumor is perceived, and of course after the heart's systole is heard or felt.

For example : let us suppose a large aneurism to exist upon the ascending aorta. At each stroke of the heart the blood would be pumped into this tumor whose walls are weaker and more yielding than the healthy arterial coats ; and not until this sac is completely filled and distended to its utmost, would the remainder of the blood be forced along into the artery beyond. The result of this would be a perceptible retardation of the arterial pulsation everywhere in both upper as well as lower extremities. In other words, there would be asynchronism between the cardiac pulse at the apex, and the arterial pulse. If the aneurism should spring from the transverse arch between the origins of the innominate and left carotid arteries, then the innominate would receive its full supply at the systole and its pulsation be unaffected. But the aneurism would then demand and receive *its* supply before the left carotid and subclavian, with a consequent retardation of the pulse throughout the distribution of those vessels. We should then have here perfect synchronism between the cardiac systole and the right upper extremity and side of the head, but asynchronism between the cardiac systole and left upper extremity as well as between the *two* upper extremities.

I am aware that there are some who claim they can detect a difference in time between the systole of the heart and the radial pulse. Of course there is a difference, and we can demonstrate it by the use of instruments for the exact measurement of time; but I maintain that this difference is too small for cognizance by human perception or consciousness. When I speak of these asynchronisms, therefore, I do not refer to these natural differences, which, as I have said, are too small for our perception.

Now let us proceed to apply these facts and this reasoning to the determination of the location of the aneurism in the case under consideration.

We find upon careful examination that, 1st, the pulsation in the tumor is synchronous with the heart systole. 2d. That the right radial pulse is entirely absent, while that of the right carotid is feeble and *retarded.* But we find, further, that the left radial and carotid is synchronous with the heart-beat. The aneurism therefore cannot spring from the aorta anywhere between its beginning and the left carotid, for if it does it would retard the pulse in this vessel as well as in the innominate. But it is the pulsation in the branches of the innominate only that is retarded, and it must be that vessel only that is involved, if our reasoning be correct; and the direction of the tumor points to the probable conclusion that it is the lower portion of this vessel that is its seat.

The pulses *each* and *all* then go to prove the aneurism to be in the lower portion of the innominate artery, and in taking that direction in which it finds least resistance, the tumor appears in the left infra-clavicular region near the manubrium of the sternum. But, you may ask, how are we to account for the dyspnœa, and aphonia, the cough and the dysphagia, of all of which this patient complains? The dyspnœa can be accounted for by the tumor pressing on the trachea, whilst the dysphagia is caused by the pressure being transmitted through the trachea to the œsophagus. The muscles of phonation, you will remember, are all, save the crico-thyroid, supplied by the recurrent laryngeal nerve, it being the motor nerve of the larynx. On the right side as it is given off from the pneumogastric it curves around the subclavian artery and ascends in the groove between the trachea and œsophagus, piercing the lower fibres of the inferior constrictor muscle, and enters the larynx close to the articulation of the inferior cornu of the thyroid with the cricoid cartilage. As a result of extended pressure on the trachea, this nerve must suffer paralysis or spasm, as was observed in this case, with the laryngoscope. Hence the aphonia and cough. It is true that we may have dyspnœa, cough, aphonia and dysphagia in aortic aneurisms, but we have absence of œdema here and furthermore, the bruit is too high to occur in the arch. You will observe that this patient is comparatively at ease while in the erect or in the left latero-prone positions, but experiences great difficulty in breathing and also coughs as soon as she assumes the dorsal or the right latero-prone positions, as a result of gravity on the tumor. After taking into consideration all of the above symptoms, the conclusion, I think, to which we are forced to arrive is, that the patient before us to-day is suffering from aneurism of the lower portion of the innominate artery.

